# Application of a novel strong promoter from Chinese fir (*Cunninghamia lanceolate*) in the CRISPR/Cas mediated genome editing of its protoplasts and transgenesis of rice and poplar

**DOI:** 10.3389/fpls.2023.1179394

**Published:** 2023-04-20

**Authors:** Shanwen Ye, Wensha Ding, Weiyuan Bai, Jiaao Lu, Linying Zhou, Xiangqing Ma, Qiang Zhu

**Affiliations:** College of Forestry, Fujian Agriculture and Forestry University, Fuzhou, China

**Keywords:** Chinese fir, promoter, protoplast, transient expression, genome editing, CRISPR/Cas

## Abstract

Novel constitutive promoters are essential for plant biotechnology. Although in angiosperms, a number of promoters were applied in monocots or dicots genetic engineering, only a few promoters were used in gymnosperm. Here we identified two strong promoters (*Cula11* and *Cula08*) from Chinese fir (*C. lanceolate*) by screening the transcriptomic data and preliminary promoter activity assays in tobacco. By using the newly established Chinese fir protoplast transient expression technology that enables *in vivo* molecular biology studies in its homologous system, we compared the activities of *Cula11* and *Cula08* with that of the commonly used promoters in genetic engineering of monocots or dicots, such as *CaM35S*, *CmYLCV*, and *ZmUbi*, and our results revealed that *Cula11* and *Cula08* promoters have stronger activities in Chinese fir protoplasts. Furthermore, the vector containing *Cas* gene driven by *Cula11* promoter and sgRNA driven by the newly isolated *CulaU6b polyIII* promoters were introduced into Chinese fir protoplasts, and CRISPR/Cas mediated gene knock-out event was successfully achieved. More importantly, compared with the commonly used promoters in the genetic engineering in angiosperms, *Cula11* promoter has much stronger activity than *CaM35S* promoter in transgenic poplar, and *ZmUbi* promoter in transgenic rice, respectively, indicating its potential application in poplar and rice genetic engineering. Overall, the novel putative constitutive gene promoters reported here will have great potential application in gymnosperm and angiosperm biotechnology, and the transient gene expression system established here will serve as a useful tool for the molecular and genetic analyses of Chinese fir genes.

## Introduction

Strong gene promoters that drive the high levels of constitutive gene expression are one of the key elements for plant biotechnology applications. Several well-known gene promoters were widely used for transgene expression in plants. The cauliflower mosaic virus *(CaMV) 35S* gene promoter is the most commonly used in dicotyledonous plants, as it strongly and constitutively drives transgene expression (Odell et al., 1985). In monocots, various promoters such as *ZmUbi1* from maize (Christensen et al., 1992), and *Act1* (McElroy et al., 1990), *OsCc1* (Jang et al., 2002), *APX*, *SCP1*, *PGD1*, *R1G1B*, and *EIF5* from rice (Park et al., 2010), are regarded as constitutive gene promoters in crop biotechnology applications. However, in gymnosperm, limited reports showed that the *CaM35S* constitutive promoter was commonly used for gene-overexpression, but its activity tends to be low in conifers (Lin et al., 2005; Song et al., 2020). Moreover, despite *CaM35S* promoter works in several conifers, there is currently a shortage of efficient promoters for high-level constitutive gene expression, which may satisfy performing multiple transgenes in a single vector that require different strong promoters in gymnosperm, as well as CRISPR/Cas mediated gene editing technology.

CRISPR/Cas mediated gene editing technology facilitates the fundamental research and molecular breeding in plants (Zhu et al., 2020; Gao, 2021). In the CRISPR/Cas system, the expression of *Cas* is generally driven by an *RNA polymerase II* (*Pol II*) promoter, while the *Pol III* promoters of small nuclear RNA (snRNA) genes, such as *U3* and *U6* promoters, are commonly used to drive sgRNA expression in plants (Zhang et al., 2017; Scheben and Edwards, 2018). Therefore, the choice of promoter is a crucial factor for *gRNA* and *Cas* gene expression in plant cells, thereby affecting the editing efficiencies (Manghwar et al., 2019; Hassan et al., 2021). Constitutive promoters sustained high expression activities in all cell types, and *CaM35S* promoter and *Ubiquitin* promoter were mostly used in the mutagenesis in dicots and monocots, respectively (Rahman et al., 2022). In gymnosperm, *CaM35S* promoter was used in-planta genome editing technologies in three conifer species, namely *P.radiata* (Poovaiah et al., 2021), *C.japonica* (Nanasato et al., 2021), and *P.glauca* (Cui et al., 2021). However, their efficiencies are quite low, probably due to the relatively low activity of *CaM35S* promoter. Optimizing those elements of CRISPR/Cas system is perquisite for the successful genome editing in plants, and identifying stronger promoters that are highly expressed in gymnosperm is crucial for its genome editing.

Since generating stable genome-edited plants is time-consuming and complex, it is necessary to develop a simple and rapid system for testing, selecting and verifying the activity of candidate promoters in its homologous system. Protoplasts transient expression assay provides a versatile tool for performing genomics, transcriptomics, metabolic and epigenetics studies (Xu et al., 2022). It nicely bypasses the difficult stable transformation procedure, and enables obtaining results within several hours or days. At present, it was widely used in studying the molecular mechanisms controlling plant growth development, plant hormone signaling, gene expression regulation, as well as other physiological processes (Eeckhaut et al., 2013; Lin et al., 2017; Priyadarshani et al., 2018; Wang et al., 2021b; Xu et al., 2022). By coupling protoplast transient expression experiments with the high-resolution imaging technology, scientists can simply, rapidly and efficiently analyze and characterize gene functions and regulatory network, such as protein subcellular localization, protein-protein interaction, transcriptional regulatory networks, as well as gene response to various external cues (Poddar et al., 2020; Wang et al., 2021a; Wang et al., 2022). To date, protoplast isolation and transfection system have been successfully used in the molecular studies in many angiosperms, such as rice (Yu et al., 2014b), switchgrass (Burris et al., 2016), barley (Yu et al., 2014a), grapevine (Zhao et al., 2016), wheat (Liao et al., 2010), ryegrass (Yu et al., 2017), Arabidopsis (Sang-Dong et al., 2007; Wu et al., 2009), maize (Sheen, 2001), tobacco (Locatelli et al., 2003), populus (Guo et al., 2012; Tan et al., 2013), cucumber (*Cucumis sativus*) (Huang et al., 2013) and pineapple (Priyadarshani et al., 2018). Mutagenesis in protoplasts can be achieved by CRISPR/Cas gene editing in several plant species (Malnoy et al., 2016; Lin et al., 2017; Liu et al., 2018; Ye et al., 2020). Whereas in gymnosperm, including Chinese fir (*Cunninghamia lanceolata* (Lamb.) Hook), the protoplast isolation and transformation techniques are still difficult.

Chinese fir, which belongs to the Cupressaceae family, covers the largest plantation area and becomes the most important timber species in China (Cao et al., 2022). With the development of modern biology, researchers are more and more interested in unraveling the molecular mechanisms as well as identifying the important genes that control wood formation, growth and development, nutrient absorption, and abiotic or biotic stress response. Currently, a large number of bioinformatical data was generated (Wan et al., 2012; Lin et al., 2020), however, few studies reported on its gene functional analysis. And until now, no native genes were transiently expressed in Chinese fir, mainly due to the lack of efficient transient and stable gene expression system. In Chinese fir, the use of native protoplast transient expression system is important as it can perform gene expression analysis without the hard technique and time-consuming problems in obtaining transgenic plants and callus, and provides an efficient, precise and consistent way for revealing molecular mechanisms. At present, a few studies reported the isolation of Chinese fir protoplasts (GeQiang and Guoning, 2012; Tang et al., 2018), which contributes to its molecular study. However, the derived protoplasts are always low yield and quality (shrunken protoplast and fractured), probably that’s why few protoplast transformation studies were reported in Chinese fir. And a more stable and easily reproducible protocol is required gene functional characterization, promoter activity assays, as well as optimization of the CRISPR/Cas elements that are suitable for its gene editing.

In this study, we identified two new promoters (*Cula08* and *Cula11*) from Chinese fir based on the transcriptomic data. To quickly check their activities natively, we first established a stable and repeatable protoplast isolation and transformation protocol enabling promoter activity assays, protein subcellular localization, protein-protein interaction, and Ribonucleoprotein (RNP)- mediated gene editing studies. With this system, we showed that promoters that commonly used in monocots and dicots transformation, such as *CmYLCV*, *ZmUbi* and *CaM35S* promoters also work in Chinese fir protoplasts. Whereas *Cula08* and *Cula11* exhibited stronger activities. More importantly, *Cula11* promoter showed much higher activity than *CaM35S* promoters in transgenic poplar, and *ZmUBI* promoter in transgenic rice, suggesting its potential application in poplar and rice genetic engineering. By using *Cula 11* promoter for the expressions of *Cas* genes (*SpRY* and *SpG*) and the newly isolated *CulaU6b* promoter for sgRNA expression, gene editing was successfully achieved in Chinese fir protoplast. The promoter identified here will contribute to the gene expression analysis in Chinese fir, as well as rice and poplar, and the protoplast-mediated transient expression system will be useful tools for molecular studies in Chinese fir.

## Materials and methods

### Plant materials and growth conditions

The Chinese fir Yangkou-020, which was widely planted in China, was used for micro-propagation. Shoot explants were excised from *in vitro* grown seedlings, and germinated on MS plus BA 0.3 mg/L, NAA 0.2 mg/L, sucrose 30 g/L and agar 6 g/L. The pH value was adjusted to 5.8. The explants grow at 26°C under 1500 lx light and 16 h/8 h photoperiod. The plantlets were sub-cultured monthly.

### Plasmid construction

For the promoter activity assays in tobacco, around 2.5-kb upstream of ATG were cloned with the primers listed in **Supplemental Table 2**. The PCR products were inserted into the upstream of *pPLV04* plasmid using in-fusion cloning methods (TaKaRa, Japan) (De Rybel et al., 2011), which enable that promoters drive the expression of the downstream eGFP. The resulted plasmids were used for premilitary promoter activity tests in tobacco.

For promoter activity assays in Chinese fir protoplasts. *35S:NLS-TdTomato* plasmid was used as the backbone (Liu et al., 2017), and the coding sequence for *TdTomato* was first replaced with eGFP between *BamHI* and *StuI* sites to generate the *35S:NLS-eGFP* plasmid. The promoters of *Cula 11*, *Cula 08*, *CmYLCV*, and *ZmUbi* were cloned by using the primers listed in **Supplemental Table 2**, and used for replacing the *CaM35S* promoter. The generated plasmids containing *Cula 11: NLS-eGFP、Cula 08: NLS-eGFP*, *YLCV: NLS-eGFP*, and *ZmUbi: NLS-eGFP* cassette were used for Chinese fir protoplast transformation, respectively.

For BiFC experiments in Chinese fir protoplast, the *CulaSCL3* and *CulaDELLA* genes were cloned with the primers listed in **Supplemental Table 2**, and fused into the *PENTRY-EN01* entry vector (Sugano et al., 2018). The fragments were further ligated into *pB4cYGW* and *pB4nYGW* vectors by gateway cloning method (Kamigaki et al., 2016), which were used for protoplast transformation.

For optimizing CRISPR/Cas elements suitable in Chinese fir protoplast, a plasmid expressing a mutant version of RFP (*muRFP*) with a base insertion at 14^th^ position was created by PCR with the *muRFP-F* and *muRFP-R* primers listed in **Supplemental Table 2**, and the resulted *muRFP* fragment was used to replace the *TdTomato* fragment at *BamHI* and *StuI* sites. A CRISPR vector containing sgRNA targeting the insertion sites of *muRFP* was designed, and was driven by Chinese fir native U6b promoter. To construct the *Cas* nucleases and *gRNA* expression plasmids, the codon-optimized *SpRY* or *SpG* gene cassettes, *35S:HPTII* expression cassettes and *Cula11* promoter were cloned with the primers listed in **Supplemental Table 2** (Sarrion-Perdigones et al., 2013; Vazquez-Vilar et al., 2016). These fragments were integrated into the *pGGP AG* vector (Decaestecker et al., 2019) to generate the intermediate plasmid containing *SpG/SpRY*-*eGFP*-*35S:HPTII* cassette by using goldengate cloning method (Sarrion-Perdigones et al., 2013). Then the *pCulaU6b-gRNA-pCula11* fragment containing the *NcoI* restriction enzyme recognition site was cloned by using sgRNA-Cas9 expression vector as templet (Zhang et al., 2014) with the primers listed in **Supplemental Table 2**. The resulted fragment was integrated into the above intermediated plasmid at *NcoI* site with in-fusion cloning method (De Rybel et al., 2011).

For constructing plasmid simultaneously expressing *RFP* and *CFP*, the *RFP-P2A-CFP* fusion gene were amplified by overlapping PCR (Fang et al., 2005) with the primers listed in the **Supplemental Table 2**, and the resulted fusion fragments were cloned into *35S:NLS-TdTomato* plasmid by replacing the *TdTomato* fragments as we described above.

For plasmids used for poplar transformation, the promoters of *CaM35S* and *Cula11* were fused into pCambia1301 vector (Yan et al., 2012) between the *HindIII* and *NcoI* restriction enzyme sites, respectively, and resulting the vectors containing promoter: *GUS* expression cassette. The sequences of all plasmids were confirmed by DNA sequencing.

### Protoplast isolation and transformation

We provided a detailed protocol on Chinese fir protoplast isolation, transient transfection, as well as RNP-mediated gene editing in the **Supplemental Method**.

### Protoplast counting

Protoplast numbers were counted with a hemocytometer (QiuJing, Shanghai, China). Forty microliters of protoplast solutions were put on the surface of the hemocytometer, and then laid the cover slide carefully. The number of intact protoplasts in the four corners of the grid was counted under microscope as described before (Wang et al., 2021a). The protoplast numbers (mL^-1^) were calculated based on the formula: the average number of the intact cells in the four corners of the gridx10^4^.

### Poplar and rice transformation

Aspen hybrid clones Yinzhong (*P. alba* x *P. berolinensis*) was used for transformation as described previously (Wang et al., 2011). Briefly, leaf explants excised from 3-week-old *in vitro* grown plants were inoculated with *Agrobacterium* strain EHA105 for 10 min, and co-cultured on MS with 0.1 mg/L NAA + 0.2 mg/L 6-BA + 50 mg/L AS for 2 days. The bacterial was removed by washing in sterilized distilled waters, and the explants were cultured on MS with 0.1 mg/L NAA + 0.2 mg/L 6-BA + 0.01 mg/L TDZ + 500 mg/L cefotaxime + 50 mg/L kanamycin. The medium should be changed every month until the kanamycin-resistant shoots were regenerated. Transgenic shoots were excised and transplanted into MS with 0.1 mg/L NAA + 500 mg/L cefotaxime + 25 mg/L kanamycin to induce the root. The whole processes were proceeded in the tissue culture room with the light intensity of around 200 µmol m^-2^ s^-1^ and photoperiod of 12 h light/12 h dark. The temperature was kept at about 25°C for the whole transformation process. After selection and molecular verification, a total of 5 independent transgenic lines were obtained for further analysis.

Rice transformation was performed following the *Agrobacterium*-mediated transformation protocol established previously (Sahoo et al., 2011). Briefly, the sterilized rice seeds were placed on the callus induction media (CIM) containing casein hydrolase (300 mg/L), proline (560 mg/L), maltose (36g/L), 2-4-D (2.5 mg/L), BAP (0.25 mg/L), and Phytagel (3 g/L) with a PH of 5.8. The induced embryogenic calli were infected with *Agrobacterium* culture and kept in co-cultivation media for 48 h. After washing, the calli were kept on selection medium containing hygromycin (50 mg/L) and cefotaxime (250 mg/L), and actively growing calli were then transferred to regeneration medium for regeneration, rooting and hardening.

### Gus staining

Histochemical staining for GUS activity in transgenic poplar and rice was performed as the protocol described in (Jefferson et al., 1987). At least 3 independent transgenic lines were used for this experiment.

### Tobacco infiltration

Tobacco infiltration was performed based on the established protocol (Yang et al., 2000). Briefly, the overnight culture of *Agrobacterium* EHA 105 containing promoter: GFP plasmids as well as the transformation helper plasmid *pSoup* was diluted and cultured to OD_600_ 0.6. The agrobacteria was harvested by centrifugation and resuspended in 10 mM MES (pH5.5) plus MS basal medium (Murashige and Skoog, 1962), and then adjusted to OD_600_ 0.5 with the acetosyringone concentration of 150 μM. The bacterial suspensions were incubated 2 h at 25°C, and then were infiltrated into leaves of 5-week-old *N. benthamiana* plants using a needless syringe. After agroinfiltration, tobacco plants were maintained in a growth chamber at 22°C under 16 h light for 48 h.

### Co-focal imaging

The incubated protoplasts were centrifuged at 900 rpm for 2 min, the supernatant was carefully removed with around 100 µL leftover. Protoplasts were resuspended with blunt tips, and transferred to slide with a cover glass for observation. Observations were used a fluorescence confocal microscope (Leica TCS SP8X DLS), adjusting for different excitation wavelengths and filter types at the 493-520 nm for GFP, 519-540 nm for YFP, 587-638 nm for RFP and 450-460nm for DAPI. The GFP fluorescence was recorded, and the green GFP signal intensities were quantified relative to using the ImageJ software (n>20).

### Genomic DNA extraction and PCR/restriction enzyme assay

Protoplasts samples were pooled and collected 2 days after plasmid or RNP transfection, the protoplast genomic DNA was extracted using TIANamp Micro DNA kit (TIANGEN, Shanghai), and PCR-amplified with high fidelity DNA polymerase using primers spanning the cutting site with the primers listed in **Supplemental Table 2**. The amplicons were then digested with a restriction enzyme that cut the wildtype sequences while not the mutant sequence that caused by CRISPR. Alternatively, *T7E1* enzyme assay were also performed for this verification (Liang et al., 2018). The products were visualized by agarose gel electrophoresis. Uncut bands were purified with TIANgel Midi Purification Kit (TIANGEN, Shanghai) according to the manufacturer’s instructions, and used as the DNA templet for the second round PCR. The 2^nd^ round restriction enzyme assay was performed again as described previously (Wang et al., 2014). The uncut bands were purified and the mutated sequences were identified by cloning and sequencing plasmids isolated from single colonies.

## Results

### Identification of novel constitutively expressed genes in Chinese fir

To screen for genes that are constitutively expressed in Chinese fir, the expression profiling was performed using the public the transcriptomic data from public database was downloaded from NCBI (SRX2586190, SRX2586189, SRX2586188, and SRX139598) (Li et al., 2017; Wang et al., 2020). These analyses identified 24 genes that were expressed in all tissues at high levels (**Supplemental Table S1**). All 24 putative promoters (~2.5 kb upstream of ATG) were cloned and inserted into the *pPLV04* vector (De Rybel et al., 2011), which resulted in constructs with each promoter linked to the green fluorescent protein gene (GFP) fused with nuclear localization signal (NLS). To make the preliminary screening easier, we first transiently expressed those constructs in tobacco. And our results showed only 4 promoters produced GFP fluorescence, *Cula11*、*Cula08*、and *Cula04* promoters have 10.8-、9.9-、and 1.5- times higher than the putative *Cula Actin* (*Cula12*) promoter based on the fluorescence signal intensities (**Supplemental Figure 1**). By checking their expressions from various tissues and growth stages, including leaves, roots, shoots, as well as seedlings at indicated days after germination, we found that the activities of *Cula11* and *Cula08* genes are constitutively expressed (**Supplemental Figure 2**). Based on the annotation, *Cula11* gene encodes a putative ubiquitin gene, and *Cula08* gene encodes a pathogenesis-related protein, whereas, their molecular functions remain to be elucidated. In summary, *Cula11* and *Cula08* promoters that might have strong activities were screened and used for the following further analysis.

### Establishment of a stable and repeatable protoplast isolation and transient expression system in Chinese fir

**Figure 1:**
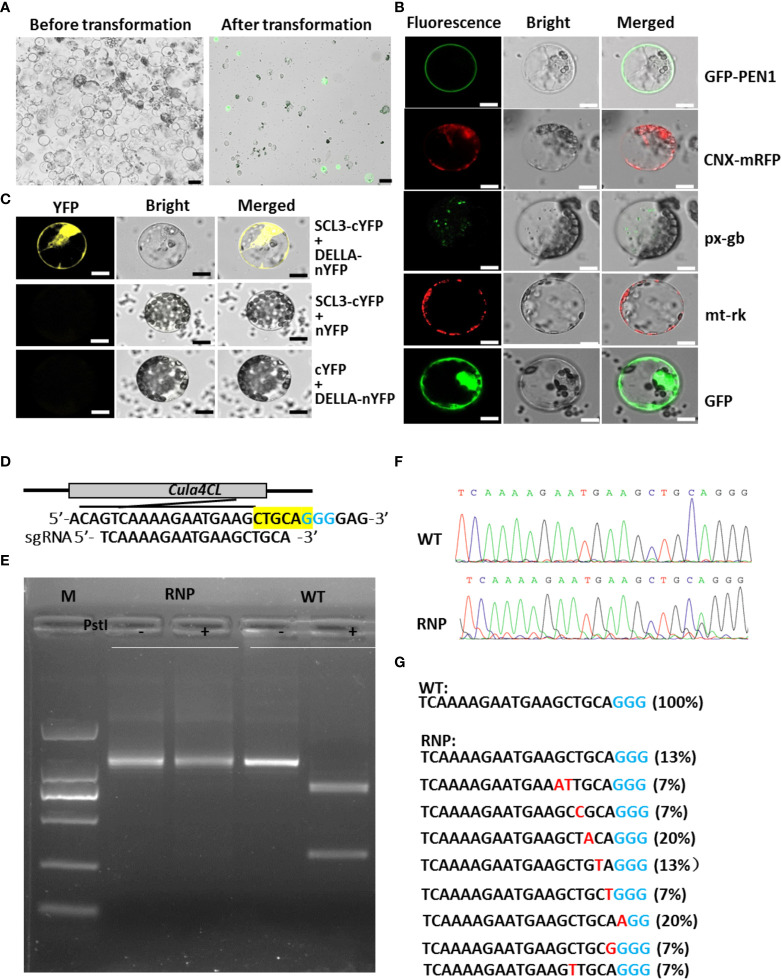
Protoplast isolation, transfection and gene function study in Chinese fir. **(A)** Status of Chinese fir protoplasts before (left) and after (right) transformation. Scale bar: 10 μm. **(B)** Subcellular localization of various cell organelle markers on plasma membrane (*PEN1-GFP*), ER (*CNX-mRFP*), peroxisome (*px-gb*), mitochondria (*mt-rk*) and control (*35S:GFP*) in Chinese fir protoplasts. Scale bar: 10 μm. **(C)** BiFC assay showing the interaction between *CulaSCL3* and *CulaDELLA* in Chinese fir protoplasts. Scale bar: 10 μm. **(D)** Schematic of the *Cula4CL* gene with the RNP targets (bottom). The corresponding PAMs (blue) and *PstI* site were highlighted (yellow). **(E)** PCR/RE assay to detect engineered RNP-induced mutations in protoplasts. **(F)** Sequencing of the PCR products from wild-type and RNP-delivered samples. **(G)** Mutation types and frequencies of RNP-delivered protoplasts. PAM (blue) and indels (Red) are shown; the ratios of certain indel type are shown in brackets.

Promoter activity assays are usually performed by analyzing the transgenic plants expressing promoter::reporter constructs. However, as for Chinese fir, it is well-known for its difficult or recalcitrant to transformation, and few promoters have been characterized in Chinese fir. Thus, alternative methods, such as protoplast-based expression system is required for functional genomics studies, including promoter activity assays. Protoplast isolation has some common features, and the enzymes used to digest the cell wall matrix usually consist of various mixtures of cellulase (Cellulase R-10 or Cellulase RS), hemicellulase, and pectinase (Macerozyme R-10). However, in different plant species, the protocols are always different (Eeckhaut et al., 2013; Lin et al., 2014). We attempted to isolate protoplasts using the reported protocols (GeQiang and Guoning, 2012; Tang et al., 2018), but found that the obtained protoplasts are always low yield and quality (shrunken and fractured) (**Supplemental Figure 3**), and not always reproducible. Then we systematically investigated various tissues including calluses, cell suspension culture, cotyledons, leaves, roots, wood-forming cells from stem and seedlings, and found that fresh aerial part of 90-day-old tissue culture plants is most suitable starting materials for its protoplast isolation (**Supplemental Figure 4**). In our lab, the enzyme recipe for releasing protoplast in Chinese fir is: using 0.4 g fresh aerial part of 1.5-month old tissue culture derived Chinese fir seedlings, incubating with 2.0% (w/v) Cellulase-RS, 1.5% (w/v) Macerozyme R-10, and 0.3% (w/v) Pectinase Y-23 for 2 h (**Supplemental Figure 5**; **Supplemental Method**). Four key steps for Chinese fir protoplast isolation and transient transformation: firstly, selection of the right starting material is crucial (**Supplemental Figure 4**; **Supplemental Method**); secondly, after transformation, a modified W5 solution with equal osmotic pressure to the MMG solution should be used, as it substantially avoids obtaining shrunken and fractured protoplasts (**Supplemental Figure 3**; **Supplemental Method**); thirdly, transient heat shock treatment (40°C for 2 min) is necessary for the successful transformation (**Supplemental Figure 5**); fourthly, Chinese fir protoplasts are quite sensitive to osmotic potential, and minute difference in the concentration of mannitol will result in different yield of intact protoplast. Under our experimental conditions, 0.5 mM mannitol provides the best results (**Supplemental Figure 5**). Compared with angiosperms, the current protoplast isolation (1.5x10^5^/g FW) and transformation (~25%-45%) efficiencies are not so high (**Figure 1A**; **Supplemental Figure 5**), but easy to operate and highly repeatable. More importantly, the protoplasts derived based on our protocol remain intact and healthy, instead of shrink and fractured shape as commonly showed in the previous protocols (**Supplemental Figure 3**), which satisfied the following gene functional studies.

To test if *CaM35S* promoter works in Chinese fir protoplasts, and the utility of the transient expression system in protein subcellular localization analysis, organelle-markers tagged with GFP or RFP, including plasma membrane marker *GFP-PEN1*(Assaad et al., 2004), endoplasmic reticulum marker *Calnexin-mRFP* (Gao et al., 2012), peroxisome marker *px-gb* and mitochondria marker *mt-rk* (Nelson et al., 2007) driven by *CaM35S* promoter were introduced into Chinese fir protoplasts, and observed under co-focal microscope. The results showed positive protoplasts display predicted organelle patterns with low background fluorescence levels (**Figure 1B**), indicating the protoplast-mediated transient expression system is applicable in analyzing protein subcellular localization in Chinese fir.

To show this transient expression system is repeatable and stable, we performed protein interaction assays. In Arabidopsis, *SCARECROW-LIKE 3 (SCL3)* protein interacts with DELLA, and acts as a positive regulator in gibberellin signaling pathway (Zhang et al., 2011). To check if the homologues of SCL3 and DELLA from Chinese fir also have interactions, *CulaSCL3* and *CulaDELLA* genes were cloned and linked with C- and N- terminal of *YFP* for bimolecular fluorescence complementation (BiFC) analysis in its protoplasts. Positive signals appear after co-transforming plasmids harboring *CulaSCL3-cYFP* and *CulaDELLA-nYFP*, while the negative controls gave no fluorescence (**Figure 1C**), supporting that SCL3 and DELLA have conserved binding ability in Chinese fir, and this transient expression system could be used for protein-protein interaction assays.

To check if this system could be used for expressing ribonucleoprotein (RNP), Cas9 protein and the *sgRNA* targeting putative *4-coumarate:CoA ligase* (*4CL*) gene (**Figure 1D**) were introduced to Chinese fir protoplasts. Protoplasts were pooled and used for PCR/restriction enzyme (RE) assay as described previously (Shan et al., 2014). The digestion-resistant bands in the RNP-treated samples were detected by PCR/RE assay (**Figure 1E**). Further cloning and sequencing of these uncut bands revealed the existence of various mutations (**Figures 1F, G**), indicating the successful native gene knock-out in Chinese fir protoplasts.

In summary, we showed that the protoplast-based expression system established here is stable and repeatable, and could be used for many molecular biology applications.

### *Cula11* and *Cula08* promoter activity assay in Chinese fir protoplast

**Figure 2:**
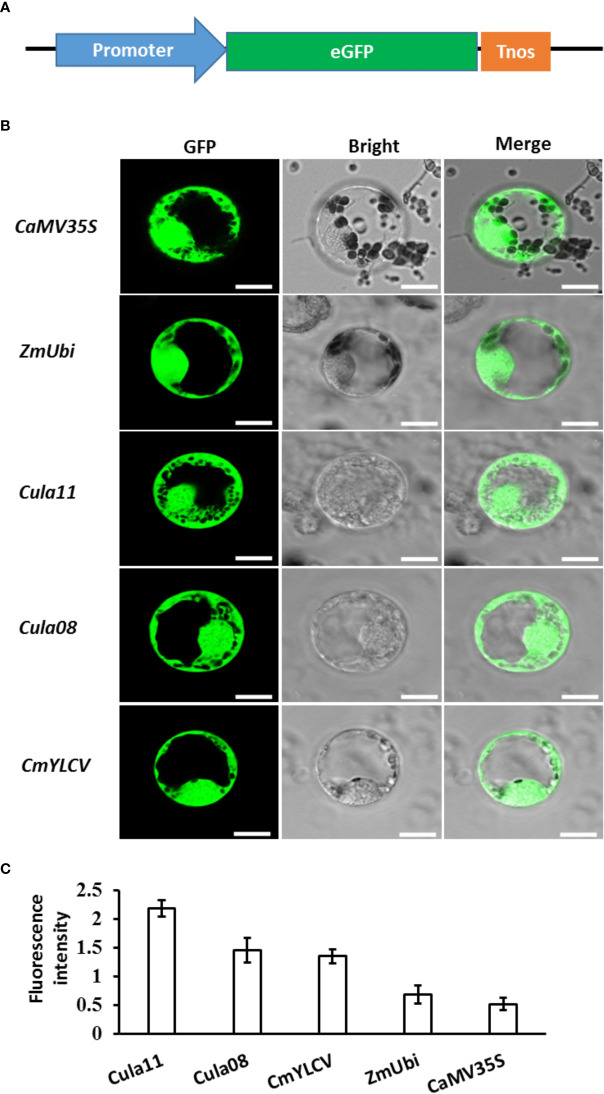
Activities of various promoters in Chinese fir protoplasts. **(A)** Schematic representation of the vector used for protoplast transformation in Chinese fir. **(B)** Represented Chinese fir protoplasts transfected with the plasmid expressing GFP driven by various promoters. Scale bar: 10 μm. **(C)** Promoter activity assays. The fluorescence of the positive protoplasts were measured using ImageJ, and the mean ± s.d for n=25 individual protoplasts for each sample were calculated. These experiments were repeated three times.

To investigate promoter activity in the Chinese fir protoplast, the well-known promoters in the genetic engineering of dicots and monocots, including *CaM35S*, *CmYLCV* (Stavolone et al., 2003), and *ZmUbi*, as well as the newly isolated *Cula11* and *Cula08* promoters, were fused with *35S:NLS-eGFP* plasmid (Liu et al., 2017), and introduced to the Chinese fir protoplast (**Figure 2A**). Our results showed that all the promoters tested here work and sufficiently drive the downstream gene expression work and sufficiently drive downstream gene expressions in Chinese fir protoplasts; however, the activities of *Cula11* and *Cula08* promoters are substantially higher than that of the promoters commonly used in angiosperms (**Figures 2B, C**). Those results suggested that *Cula11* and *Cula08* promoters have strong strength in Chinese fir protoplasts, and they might be suitable for driving high levels of gene expression in stable transformants of Chinese fir in the future.

### *Cula11* and *CulaU6b* promoter contribute to the CRISPR/Cas mediated gene editing in Chinese fir protoplast

**Figure 3:**
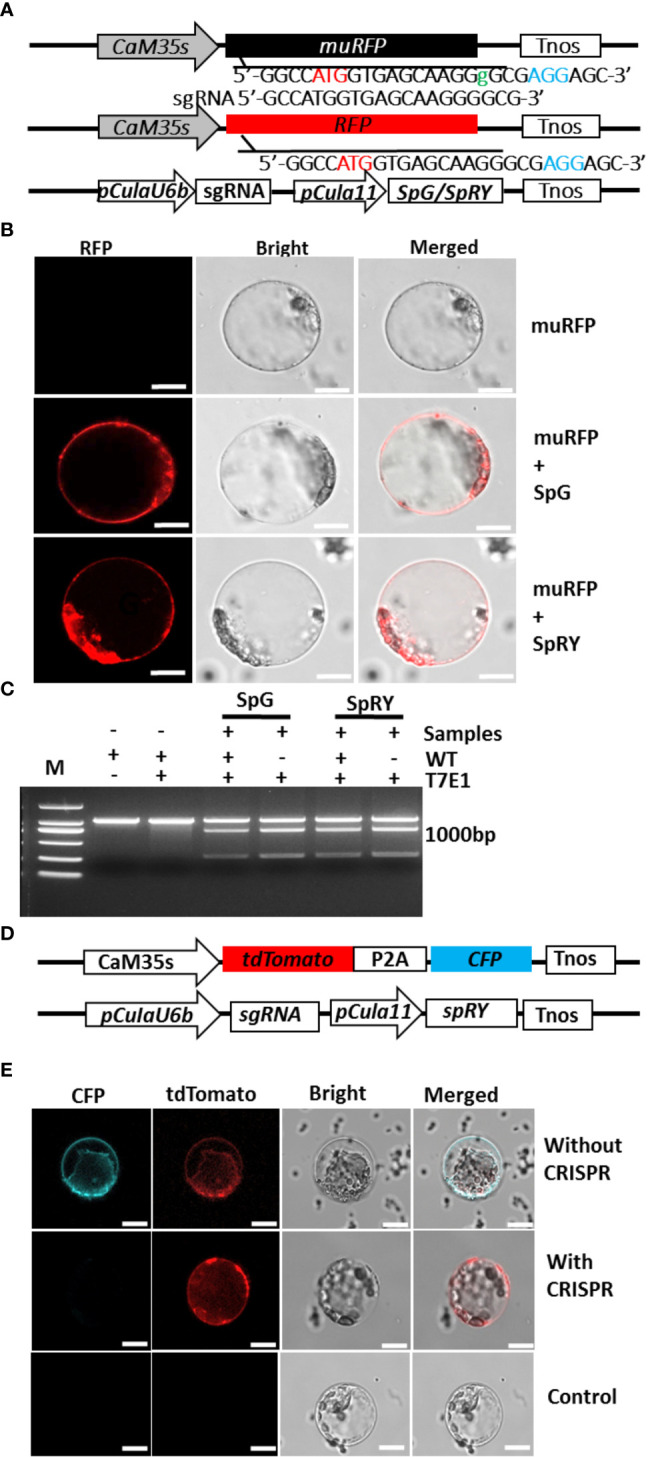
Application of *Cula11* promoter in the CRISPR/Cas mediated genome editing in Chinese fir protoplasts. **(A)** Strategies for verifying CRISPR/Cas constructs in Chinese fir protoplasts. Constructs expressing non-functional *muRFP* (Top) and optimized CRISPR elements (Bottom) were co-transformed, and *muRFP* will be corrected to *RFP* once the CRISPR elements work (Middle). **(B)** Representative Chinese fir protoplasts expressing *muRFP* (Top), and co-transfected with *SpG* and *SpRY* expressing constructs that reproducibly emitting fluorescence signals (Middle and Bottom),Scale bar: 10 μm. **(C)** Detecting of target mutations by T7 endonuclease (*T7E1*) assay. **(D)** Alternative strategy for verifying *CRISPR*/*Cas* constructs in Chinese fir protoplasts. The *tdTomato* and *CFP* simultaneously expressed under *CaM35S* promoters (Top), and CRISPR plasmid which *sgRNA* was driven by *pCulaU6b* and *SpRY* driven by *pCula11*. **(E)** Detection of gene mutation in Chinese fir protoplasts. Representative protoplasts simultaneously expressing *CFP* and *tdTomato* without CRISPR plasmid (Top), and CFP signal disappeared when optimized CRISPR elements work (Middle), protoplasts that give no fluorescence were used as control (Bottom). Scale bar: 10 μm.

CRISPR/Cas technology becomes revolutionary tool for plant molecular breeding (Zhu et al., 2020). However, no gene editing was achieved in Chinese fir. To select the proper CRISPR/Cas elements that function in its gene editing, a mutant RFP version (*muRFP*) containing 1-bp “Guanine” insertion and giving no fluorescence was artificially constructed (**Figure 3A**). The CRISPR/Cas elements composed of plant codon-optimized *SpRY* or *SpG* gene (Ren et al., 2021) driven by the *Cula11-*strong promoter, and the sgRNA driven by *CulaU6b* promoter (**Supplemental Figure 6**) were co-transformed with this plasmid, and once CRISPR/Cas system works in Chinese fir, RFP function might be restored by deleting the additional “Guanine” in *muRFP* sequence (**Figure 3A**). After co-transformation, RFP signals appeared in both *SpRY* and *SpG* expressing protoplasts (**Figure 3B**), indicating the CRISPR/Cas elements work in Chinese fir protoplasts and *muRFP* sequence was successfully edited to *RFP* coding sequence. Further PCR/RE assay confirmed the effectiveness of these CRISPR elements in Chinese fir (**Figure 3C**). Since the current protoplast isolation and transformation efficiencies are not so high, and to make the test more convenient, a plasmid simultaneously expressing *tdTomato* and *CFP* that produce both red and blue signals was co-transformed with CRISPR/Cas plasmid targeting the CDS of *CFP*, a small proportion of protoplasts gave only red but not blue fluoresce were observed under confocal microscopy (**Figures 3D, E**), further confirmed that the *Cula11* promoter could be used for CRISPR/Cas mediated gene editing in Chinese fir protoplast.

### *Cula11* promoter showed stronger activity in transgenic poplar and rice

**Figure 4:**
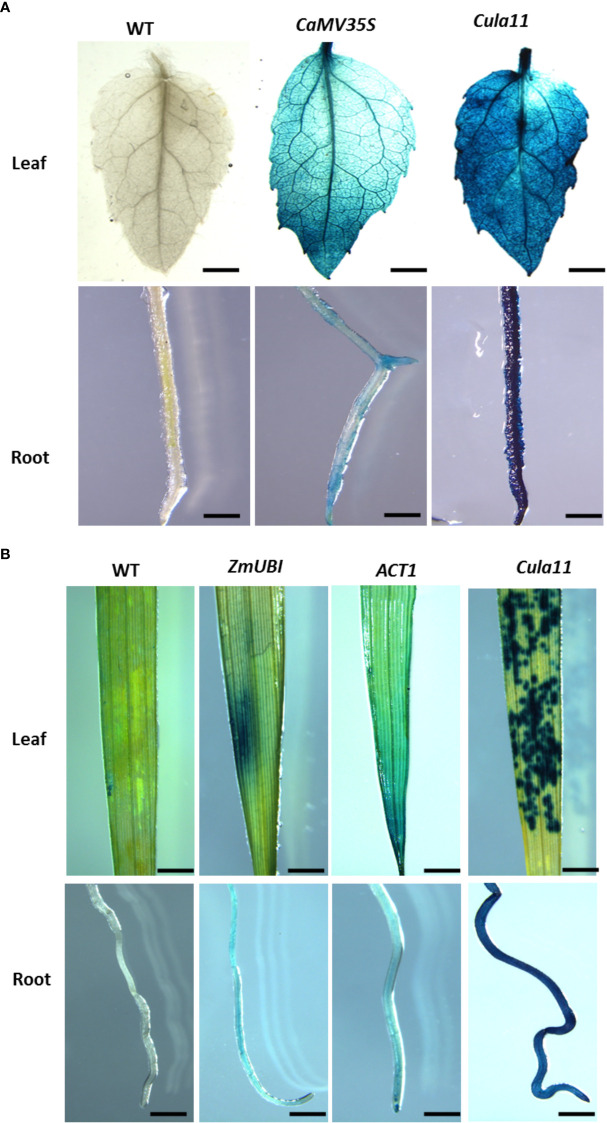
*Cula11* promoter activity assay in the root and shoot of the *promoter:GUS* transgenic poplars and rice. **(A)** Transgenic poplars harboring the *Cula11 promoter:GUS* were analyzed in comparison with the transgenic lines containing *35S:GUS* constructs. Roots (UP panel) and shoots (Bottom Panel) from at least 3 independent transgenic lines were analyzed, and represented images were shown here. Scale bar: 2 cm; **(B)** Transgenic rice harboring *Cula11 promoter:GUS* were analyzed in comparison with the transgenic lines driven by *ZmUbi : GUS* and *Act1:GUS*. Roots (UP panel) and shoots (Bottom Panel) from at least 3 independent transgenic lines were analyzed, and represented images were shown here. Scale bar: 1 cm.

To check if *Cula11* also works in other plant species, we generated transgenic poplar expressing *35S::GUS* and *Cula11 promoter::GUS*, and compared their activities by GUS staining. The results showed that *Cula11* promoters have much stronger activities in both leaves and roots compared with *35S* promoter, as revealed by GUS staining signal (**Figure 4A**). Moreover, to check if it also works in monocot such as rice, we also generated GUS expressing lines driven by *ZmUBI*, *ACT1* and *Cula11* promoters, and compared their activities in transgenic rice. Our results also supported that *Cula11* promoter has much stronger activities than the commonly used *ZmUBI* and *ACT1* promoter (**Figure 4B**). In summary, the *Cula11* promoter is useful as a promoter for ubiquitous expression of transgene in both monocots and dicots.

## Discussion

Despite isolating numerous promoters from numerous varieties of plants, only a few of them are commonly used in plant genetic engineering. Moreover, the promoter that could be used in gymnosperm is still limited. Up to now, only a few reports have shown that *CaM35S* promoter works in conifers (Tzfira et al., 1996; Liu et al., 2020; Poovaiah et al., 2021), and isolating novel promoters that are potentially used for its genetic engineering is important. In this report, we screened two strong promoters from Chinese fir, and by using the newly established protoplast mediated transient gene expression system, we showed its strong activities natively. More importantly, *Cula11* promoter has much stronger activity in transgenic poplar and rice, suggesting its potential broad usage in plant genetic engineering.

Due to the technical obstacles and the long time required in generating stable transgenic Chinese fir, an easy and repeatable transient expression system is urgently required for accelerating plant genomic study, including promoter activity analysis. In this report, we showed that generating high-quality protoplasts depends on the proper shoot tip materials, the composition of the enzyme solution, as well as the compositions of modified W5 solution for incubation (**Supplemental Method**). In the preliminary experiment, we tested the protoplast separation efficiency from various tissues of Chinese fir, including cotyledon, leaves, and callus. However, quite low efficient and low-quality protoplasts were obtained in most cases when compared with the fresh aerial part of 90-day old tissue culture plants. This protocol can be used in different Chinese fir varieties that were widely planted in China, including Chinese fir 020 and 061. Notably, we found that the Chinese protoplasts are quite sensitive to the osmotic stress. Protoplasts are easily shrunken under high osmotic potential, while burst under low osmotic potential. The compositions of modified W5 solution, which has equal osmotic pressure with the following MMG solution, is quite important to keep the Chinese fir protoplast intact. Moreover, we showed that the protoplasts prepared from this method are healthy and competent for transformation, and successful transformation not only depends on the PEG concentration and the amount of DNA, but also heat-shock treatment (**Supplemental Figure 5**). The protocol provided here will be a convenient technique for promoter function validation, as well as many other molecular biology studies in Chinese fir.

With the advance in DNA sequencing technology, the bulk of transcriptomic data of Chinese fir were generated, providing good chance for studying the gene expression patterns of different tissues and screening genes that have high expression in all tissues. In this study, 24 promoters were cloned for the preliminary promoter activity assays in tobacco (**Supplemental Figure 1**). Further test in Chinese fir protoplasts showed that both *Cula11* and *Cula08* promoters have strong activities, much higher than the commonly used promoter in monocots and dicots, such as *CaM35S*, *ZmUBI*, or *CmYLCV* (**Figure 1**). And those two promoters, especially *Cula11* that with stronger activity than *Cula08*, have the great potential for Chinese fir transformation. At present, although the *Cula11* gene was annotated as ubiquitin, its molecular functions remain unknown. In the future, this gene should be characterized at the cellular level. Several ubiquitin promoters that derived from multiple plant species, including Arabidopsis, potato, sunflower, rice or maize, were isolated, but most of them were not frequently applied (Sharma and Sharma, 2009). The most widely applied constitutive promoter utilized for genetic engineering were *ZmUBI* from maize (Cornejo et al., 1993). To the best of our knowledge, *Cula11* promoter is the only one that was isolated from gymnosperm, and has the potential for gymnosperm and angiosperms genetic engineering.

CRISPR/Cas mediated genome editing technology provides a powerful tool for molecular study and breeding in Chinese fir. High levels expression of *Cas* gene is perquisite for the successful gene editing in Chinese fir. By using the newly isolated *Cula11* promoter, we showed that CRISPR/Cas mediated gene editing was successfully achieved (**Figure 3**), indicating its potential usage in future genetic engineering.

With the progress of gene stacking technology (Zhu et al., 2017), a battery of different promoters are needed to avoid homology-dependent gene silencing that commonly happens in transgenic plants with multiple copies of the same promoter (Potenza et al., 2004). Here we showed that the novel gene promoters from Chinese fir confer high levels of gene expression in transgenic rice and poplar, suggesting its potential alternative to *35S* promoter and *ZmUBI* promoters for the high-level expression of genes in monocots and dicots. In the future, expression patterns of these genes during the whole spectrum of transgenic plant growth, especially in rapidly dividing cells, should be functionally analyzed.

In conclusion, we reported two useful promoters for high-level expression of genes in gymnosperm such as Chinese fir, as well as in angiosperms such as rice and poplar. We established a repeatable protocol and highlighted the tips for protoplast isolation and transfection in Chinese fir, and for the first time we showed that this transient expression system satisfied its gene functional studies in Chinese fir protoplast, including protein localization, protein-protein interaction assays and Ribonucleoprotein (RNP) - mediated gene editing studies. Moreover, we showed the activities of several well-known promoters in Chinese fir protoplasts, and highlighted two new native stronger promoters that potentially be used for the genetic engineering of both poplar and Chinese fir in the future. With the newly isolated *Cula11* and *U6b* promoters, CRISPR/Cas-mediated gene editing was successfully achieved in its protoplast. The technology provided here will facilitate genomic study and genetic engineering in Chinese fir in the future.

## Data availability statement

The original contributions presented in the study are included in the article/**Supplementary Material**, further inquiries can be directed to the corresponding author/s.

## Author contributions

XM and QZ conceived this project, designed experiments, and interpreted the results. SY, WD, WB, JL and LZ performed the experiments and analyzed the data. All authors read and approved the submission of this manuscript. All authors contributed to the article and approved the submitted version.
