# Supplementary figures and images for: Application of a novel strong promoter from Chinese fir (Cunninghamia lanceolate) in the CRISPR/Cas mediated genome editing of its protoplasts and transgenesis of rice and poplar
Source: Front Plant Sci. 2023 Apr 20;14:1179394. doi: 10.3389/fpls.2023.1179394 (PMC10157052; doi:10.3389/fpls.2023.1179394)

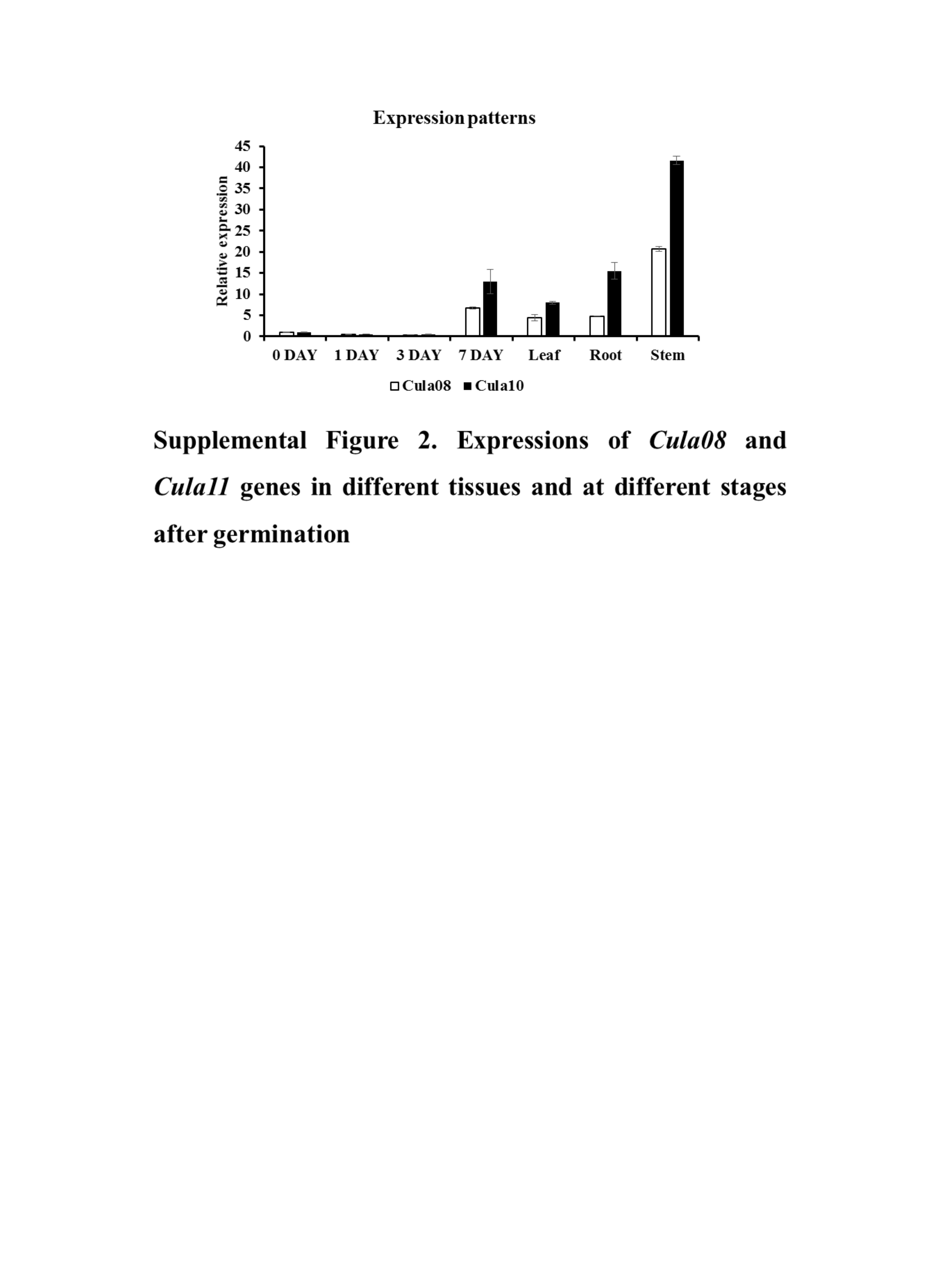

Supplement: Supplementary file 2 [file Image_2.jpg]

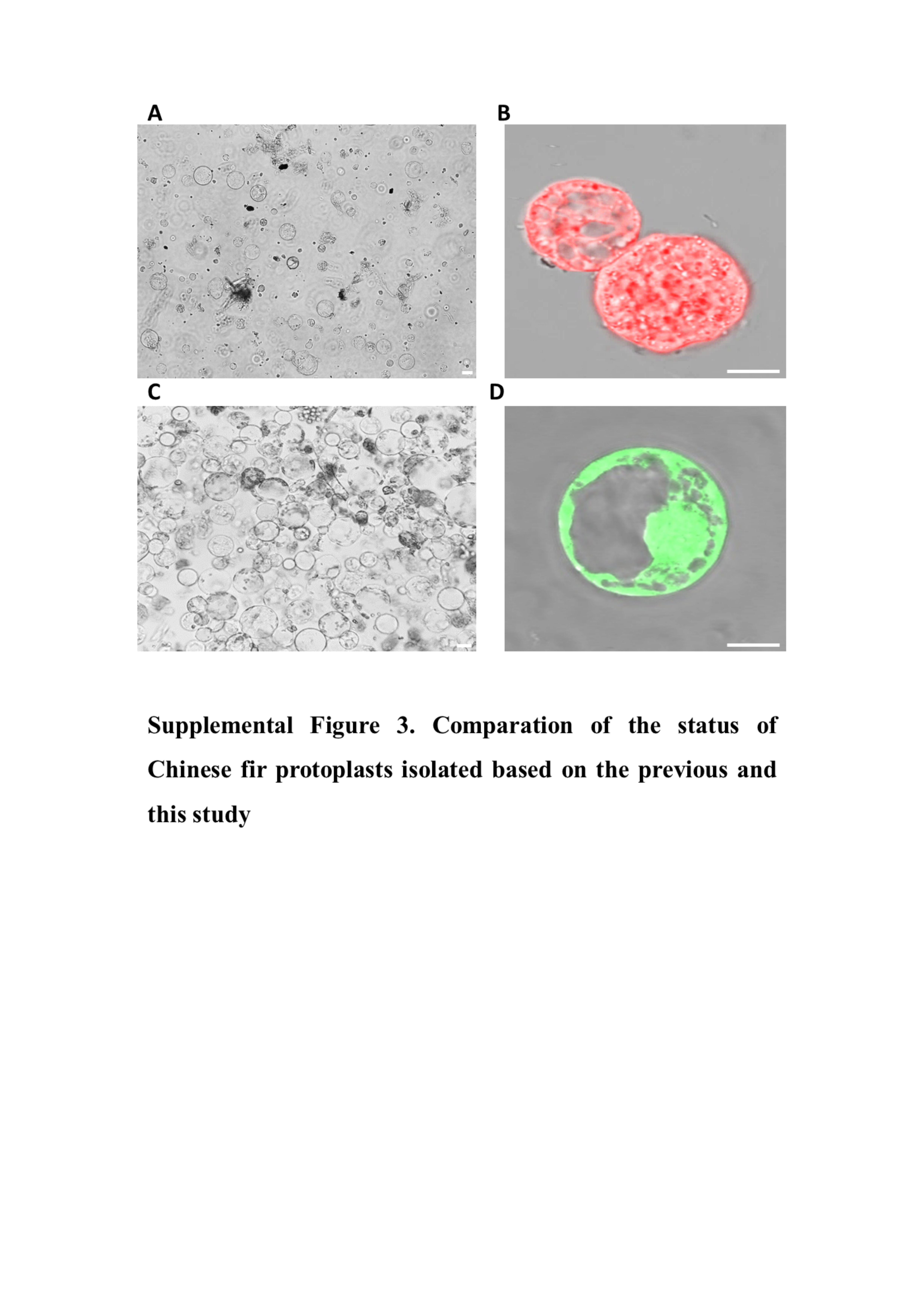

Supplement: Supplementary file 3 [file Image_3.jpg]

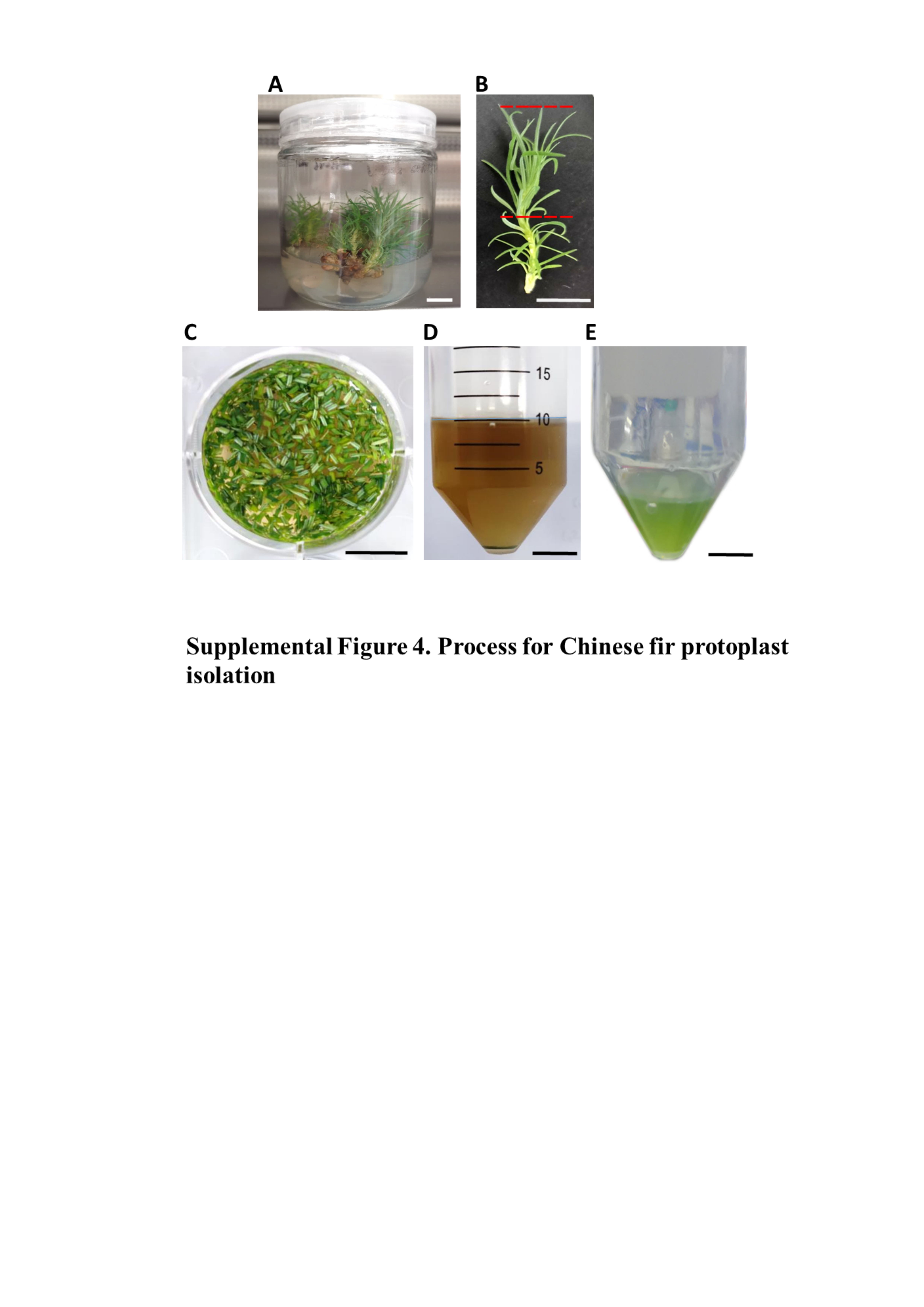

Supplement: Supplementary file 4 [file Image_4.jpg]

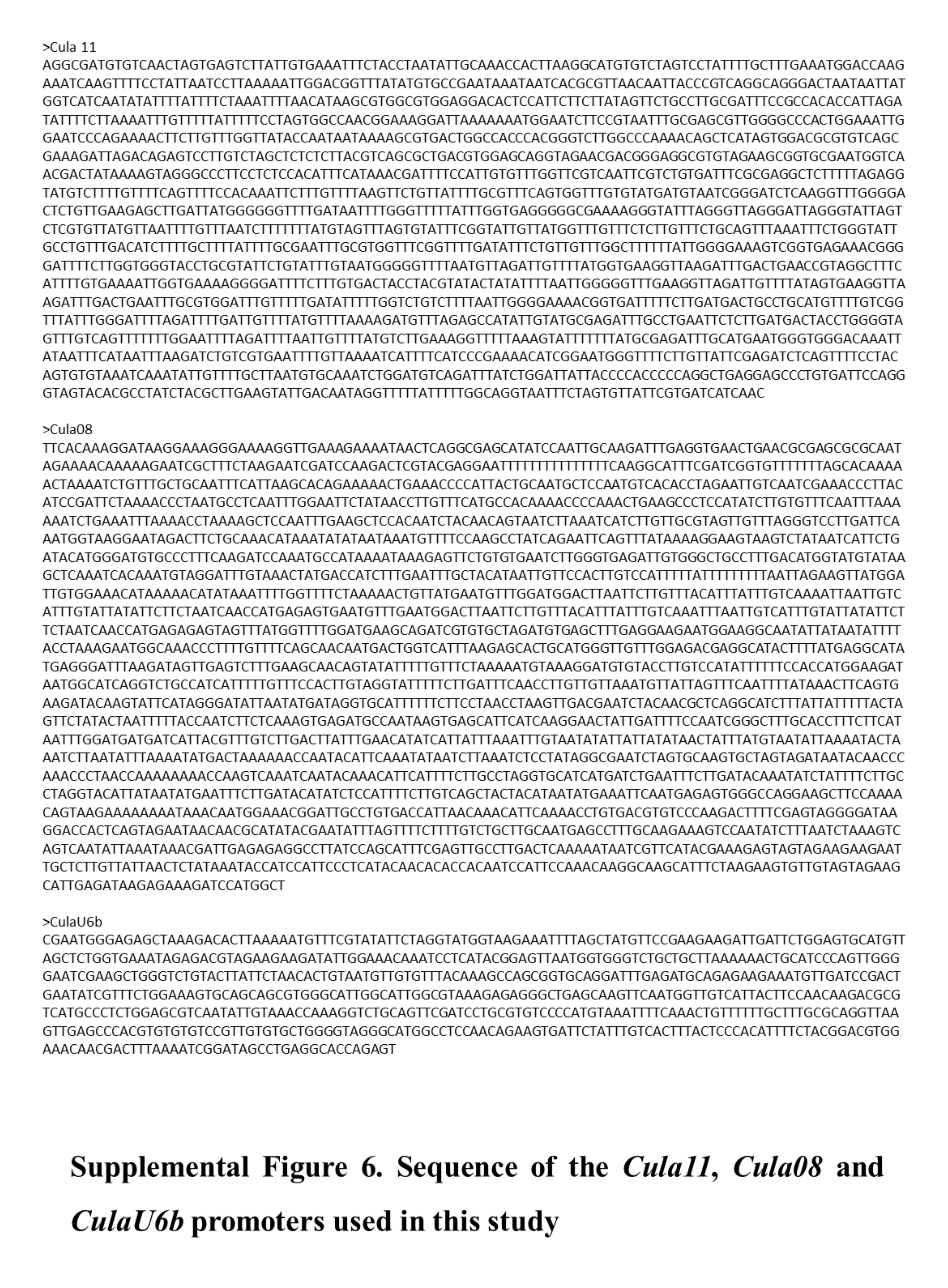

Supplement: Supplementary file 6 [file Image_6.jpg]
