# Supplementary material for: Application of a novel strong promoter from Chinese fir (Cunninghamia lanceolate) in the CRISPR/Cas mediated genome editing of its protoplasts and transgenesis of rice and poplar
Source: Front Plant Sci. 2023 Apr 20;14:1179394. doi: 10.3389/fpls.2023.1179394 (PMC10157052; doi:10.3389/fpls.2023.1179394)
Supplement: Supplementary file 7 [file DataSheet_1.docx]

**Plant growth:**

Young shoots of Chinese fir (Ecotype: Yangkou 020) were grown in the medium (MS+BA 0.3 mg/L+ NAA 0.2 mg/L+ sucrose 30 g/L+ agar 6 g/L, PH=5.8) and incubated at 26 °C under 1500 lx light, 16 h/8 h photoperiod. The medium should be changed every month.

**1. Step-by-step method details**

**1.1. Protoplast isolation**

TIMING: ∼3.5-4 hrs

1. Material selection: The newly emerged buds were cultured for 90 days, and healthy shoot apex with fully expended leaves should be carefully selected (Supplemental Figure 2).
2. Add 5 mL prepared enzyme buffer into the 85 mm x128 mm x 20 mm cell culture petri dish.
3. Carefully fold the sample. Slice the sample into 1.5-2 cm strips using a sharp surgical blade.

**Critical:** The proper stage of the sample is crucial for the successful protoplast isolation. The apex of 90-day old seedlings is suitable for digestion. Slicing the sample horizontally instead of vertically using a sharp surgical blade. Avoid crushing the edge of the cell, and causing excessive cell rupture.

1. Submerged 0.4 g samples into the 5 mL enzyme buffer, treated the sample under vacuum ∼100 mbar for 10 mins.

**Critical:** Too much materials reduce the yield of protoplasts. Based on our experience, 0.4 g material submerging in 5 mL enzyme buffer gave the best results. Be sure all samples are submerged into the enzyme buffer. Vacuum should be released slowly to avoid plant cell damage.

1. Fix the petri dish on a shaker at a speed of 90 rpm and 26 ˚C for 2 hrs.

**Critical:** The enzymatic hydrolysis temperature should be 26 ℃. The enzymatic hydrolysis temperature will lead to poor results if the temperature is too low. The incubation time affects the quality of protoplasts. Normally, two-hour digestion is enough, and the enzymatic hydrolysis time can be appropriately extended to 2.5-3 hrs, but should not exceed 3 hrs. Gently stir the hydrolysate per hour, carefully remove the wax-like substance floating on the surface of the liquid.

1. Add 5 mL modified W5 buffer to the protoplast mixture to stop the reaction.

**Critical:** The modified W5 compositions is crucial to avoid obtaining shrunken protoplasts. Be sure to gently operate when mixing or shaking the samples, any violent movement of the solution will reduce the yield of intact protoplasts. The modified W5 Buffer should be cooled down on ice for at least 30 min.

7. Wash the 40 μm cell strainer with 5 mL the modified W5 buffer twice, put the cell strainer on a 50 mL falcon tube, carefully transfer the protoplast mixture onto the cell strainer using blunt tips to filter the protoplast.

**Critical:** Blunt tips should be used to avoid penetrating the protoplasts. Be sure to operate very gently.

1. Collect the protoplast by centrifuging at 26 ˚C, 900 rpm for 2 mins, remove the supernatant as much as possible without touching the pellet.

**Critical:** If possible, reduce the centrifuge acceleration rate.

1. Resuspend the protoplast with 3 mL modified W5 buffer by soft pipetting the pellet with a blunt tip.

**Critical:** Be sure to operate very slowly and gently.

9). Place the tube containing protoplast vertically on ice for 30 min, collect the protoplast by centrifuging at 26 ˚C, 900 rpm for 2 mins.

10). Resuspend the protoplast with MMG buffer up to 2.0ⅹ10^5^ cells/ml.

**Critical：**Based on our experience, resuspension the protoplasts in around 800 µl MMG solution will lead to a concentration of around 2.0ⅹ10^5^ cells/ml.

11). Place the protoplast on ice and proceed to the experiment.

**1.2 Protoplast transformation**

TIMING: ∼45 mins

1. Mix 10 μL plasmids (2 μg/μL) in an appropriate ratio thoroughly in a 1.5 mL Eppendorf tube.

**Note:** If two plasmids need to be transformed, equal amounts of plasmids should be mixed in advance. The plasmids should be prepared freshly.

1. Add 200 μL prepared protoplasts, mix gently with a blunt tip.

**Critical:** Mix gently and wait for 2 min.

1. Add 220 μL PEG solution to the DNA-protoplasts, mix the mixture by slowly inverting the tubes 6~8 times.

**Note:** The PEG solution should be prepared freshly.

1. Heat shock the DNA-protoplasts-PEG mixture at 40 ˚C in a water bath for 2 mins. Then incubate for 3 minutes at room temperature.

**Critical:** Heat shock treatment in the water bath is crucial for the successful transformation in Chinese fir protoplast, incubation on ice after heat shock is optional.

1. Add 500 μL modified W5 buffer to the transformation mixture gently, mix the sample by slowly inverting the tube.

**Critical:** The modified W5 solution should be used.

1. Collect the protoplasts by centrifuging at 26 ˚C, 900 rpm for 2 min, remove the supernatant completely by careful pipetting without disturbing the pellet.

**Critical:** When the reaction is terminated by W5 solution, the solution is relatively thick. Do not absorb the floating protoplasts and cause loss. Leave some liquid to avoid protoplast loss.

1. Repeat step 13、14 by using 750 μL W5 buffer.
2. Resuspend the protoplasts with 750 μL W5 buffer by very soft pipetting with a blunt tip.
3. Coat the wells in 12-well tissue culture plates with 10% BSA. Add 750 μL modified W5 buffer each.
4. Transfer the cell suspension into the tissue culture plate and culture the protoplasts at 24 ˚C for 24-48 hrs in the dark.

Note: The volume of each sample is 1.5 ml.

**1.3 RNP transformation**

TIMING: ∼45 mins

1. Mix 4 µl GXL 5X Buffer；20 µg gRNA；40 µg of bNLS-Cas9 protein, and make final volume of 20 µl in a 1.5 mL Eppendorf tube, incubate at 37 °C for 30 min.

**Critical:** It is important to use freshly assembled products for each experiment.

1. Add 200 μL protoplasts prepared from step 11, mix gently with a blunt tip, and wait for 2 min at room temperature.
2. Add 220 μL PEG4000 solution to the protein-protoplasts, slowly inverting the tubes 6~8 times.
3. Incubation for 30 minutes at room temperature.
4. Add 500 μL modified W5 buffer to the transformation mixture gently, mix the sample by slowly inverting the tube.
5. Collect the protoplasts by centrifuging at 26 ˚C, 900 rpm for 2 min, remove the supernatant completely by careful pipetting without disturbing the pellet.
6. Resuspend the protoplasts with 750 μL modified W5 buffer by soft pipetting with a blunt tip.
7. Coat the wells in 12-well tissue culture plates with 10% BSA. Add 750 μL W5 buffer each.
8. Transfer the cell suspension into the tissue culture plate and culture the protoplasts at 24 ˚C for 48 hrs in the dark.

10）At the end of culture, protoplasts were collected and total DNA was extracted for cloning of target fragments.

**Critical:** Multiple samples can be pooled for DNA extraction by using TIANamp Micro DNA KIT (TIANGEN,Cat.#DP316)

**2. Troubleshooting**

**Problem 1: Low protoplast yield**

- 1.5~2 cm fresh and healthy aerial part of 90-day old tissue culture plants should be selected as starting materials.
- Slice the material horizontally instead of vertically, and make sure the materials were finely sliced.
- The chopped leaf material should be quickly put into the enzymatic hydrolysis solution, extend the enzyme digestion to 3 hrs if necessary.
- The concentration of mannitol in enzymolysis solution can be adjusted from 0.4 M to 0.6 M, make sure all the buffers and make sure the key buffers are sterile and made fresh.

**Problem 2: Shrunken and fractured protoplasts with cell debris**

- The modified W5 solution is vital for avoid obtaining shrunken and fractured protoplasts.
- Select the right material, and always slice the sample with the sharp surgical blade and avoid any kind of crushing of the samples.
- Always use blunt tips and resuspend the cells very gently as indicated in the protocol.
- Make sure the centrifuge speed is lower than 900 rpm, and the vacuum accelerate slowly.
- After 48 hours’ culture, the aggregated fragments in the solution should be removed carefully before protoplast collection.

**Problem 3: Low transformation rate**

- The PEG solution should be freshly prepared before use, and try to dissolve PEG completely by vertexing.
- Heat shock treatment is a key step for the successful transformation experiments. However, it is optional to place the protoplasts in ice-water bath or leave it in the room temperature after heat shock.
- The plasmid quality is important for a successful transformation experiment.
- In some cases, positive signals might be observed 24 hours after transformation.

3. **Solutions**

1) Enzyme solutions

Dissolve 0.1 g cellulase RS, 0.075 g macerozyme R10, 0.015 g pectinase in 3 ml ddH_2_O, add 50 µL 2 M KCl, 500 µL 0.2 M MES and 0.545 g mannitol to the mixture in the order specified. Heat up the mixture to 55 ˚C for 10 mins in a water bath to deactivate the proteases and cool down the mixture to room temperature. Add 50 µL 1 M CaCl_2_ and 150 µL 10% BSA into the buffer and mix gently. Finally, add ddH_2_O up to 5 ml.

| Reagent | Final concentration | Amount |
| --- | --- | --- |
| cellulase RS | 2% | 0.1 g |
| macerozyme R10 | 1.5% | 0.075 g |
| pectinase | 0.30% | 0.015 g |
| ddH_2_O |  | 3 ml |
| 2 M KCl | 20 mM | 50 µL |
| 0.2 M MES | 20 mM | 500 µL |
| mannitol | 0.6 M | 0.545 g |
| 55 ˚C for 10 mins in water bath then cool down to room temperature | | |
| 1 M CaCl_2_ | 10 mM | 50 µL |
| 10% BSA | 0.3% | 150 µL |
| ddH_2_O |  | up to 5 mL |

**Note:** Sterilize the buffer using 0.22 µm filters in a laminar hood. Fresh prepare the solutions before use.

**2) Prepare modified W5 Buffer (500 mL)**

The osmotic pressure of W5 was modified to equal to the MMG solution based on the formula π=cRT （π: osmotic pressure; C: molarity; R: gas constant; T: temperature (K)）. Mix 2.34 g NaCl, 9.19 g CaCl_2_•2H_2_O, 0.15 g MES, 0.185 g KCl with ddH_2_O and then fill up to 500 mL with ddH_2_O. Autoclave at 121 ˚C for 20 mins. Stored at 4 ˚C up to 4 weeks.

| Reagent | Final concentration | Amount |
| --- | --- | --- |
| NaCl | 80 mM | 2.34 g |
| CaCl_2_·2H_2_O | 125 mM | 9.19 g |
| MES | 0.03% | 0.15 g |
| KCL | 5 mM | 0.185 g |
| ddH_2_O |  | up to 500 mL |

**Note:** W5 solution should be autoclaved at 121 ˚C for 20 mins.

**3) Protoplast transformation buffers**

- **Prepare** **MMG Buffer (500 mL)**

Mix 1.525 g MgCl_2_•6H_2_O, 0.5 g MES, 36.5 g mannitol with ddH_2_O up to 500 mL. Autoclave at 121 ˚C for 20 mins. Stored at 4 ˚C up to 4 weeks.

| Reagent | Final concentration | Amount |
| --- | --- | --- |
| MgCl_2_·6H_2_O | 15 mM | 1.525 g |
| MES | 0.1% | 0.5 g |
| mannitol | 0.4 M | 36.5 g |
| ddH_2_O |  | up to 500 mL |

**Note:** MMG Buffer should adjust pH to 5.6 with KOH.

- **Prepare 40% PEG solution**

Dissolve 1 g PEG4000 with 625 μL 0.8 M mannitol, 250 μL 1 M CaCl_2_ and add ddH_2_O up to 2.5 mL. Mix thoroughly by vertexing.

| Reagent | Final concentration | Amount |
| --- | --- | --- |
| PEG4000 | 40% (m/v) | 1 g |
| 0.8 M mannitol | 0.2 M | 625 μL |
| 1 M CaCl_2_ | 0.1 M | 250 μL |
| ddH_2_O |  | up to 2.5 mL |

**Note:** PEG solution should be freshly prepared, normally 30 mins before the experiment. Be sure that PEG4000 is completely dissolved without any bubbles.
